# Frailty in chronic myeloid leukemia: evidence from 2016–2018 Nationwide Inpatient Sample of the US

**DOI:** 10.1186/s12877-023-03962-7

**Published:** 2023-05-30

**Authors:** Lin Huan-Tze, Liu Yun-Ru, Lee Kuan-Der, Tzeng Huey-En

**Affiliations:** 1 Department of Internal Medicine, Division of Hematology and Oncology, Taipei Medical University Hospital, Taipei, Taiwan; 2 Joint Biobank, Office of Human Research, Taipei Medical University, Taipei, Taiwan; 3 Department of Medical Research, Taichung Veterans General Hospital, 1650 Taiwan Boulevard Sect. 4, Taichung City, 40705 Taiwan; 4 Department of Medicine, Division of Hematology/Medical Oncology, Taichung Veterans General Hospital, Taichung City, Taiwan; 5 Ph.D. Program for Cancer Molecular Biology and Drug Discovery, and Graduate Institute of Cancer Biology and Drug Discovery, College of Medical Science and Technology, Taipei Medical University, Taipei, Taiwan; 6 Department of Post-Baccalaureate Medicine, College of Medicine, National Chung Hsing University, Taichung, Taiwan

**Keywords:** Frailty, Chronic myeloid leukemia (CML), In-hospital outcome, Nationwide Inpatient Sample (NIS)

## Abstract

**Background:**

Frailty is a marker of poor prognosis in older adults with hematologic malignancies and contributes to the severe vulnerability of the aging population to adverse health outcomes. This study aimed to determine the association between frailty and outcomes in hospitalized patients with chronic myeloid leukemia (CML).

**Methods:**

The International Classification of Diseases (ICD-10) identified data on hospitalized patients 20 years or older admitted with CML between 2016 and 2018 in the US National Inpatient Sample (NIS) database. The cohort was further divided into groups of patients with or without frailty. Logistic regression analysis was performed to determine associations between study variables and clinical outcomes. A stratified analysis of the association between frailty and in-hospital mortality by age group was also performed.

**Results:**

A total of 13,849 hospitalized patients with CML were included, 49.6% of whom had frailty. The mean age of the patients was 65.1 years, and 7,619 (56.2%) of them were male. Frailty was associated with nearly 4 times the risk of in-hospital mortality, 3 times the risk of unfavorable discharge, 3 times the risk of prolonged LOS,, and significantly more in total hospital costs. In addition, frailty was associated with a significantly increased risk of in-hospital mortality in all age subgroups (< 40 years, 40–59 years, and > 60 years) compared with no frailty.

**Conclusions:**

Frailty strongly predicts poor clinical outcomes in US patients with CML.

**Supplementary Information:**

The online version contains supplementary material available at 10.1186/s12877-023-03962-7.

## Background

The hematologic malignancies are mostly and increasingly diagnosed in older adults [1-4]. However, although age is strongly associated with malignant hematologic diagnoses, it might not precisely reflect the condition of individual patients. Therefore, several tools and assessments from the geriatrics discipline are being incorporated into routine oncology care. Frailty has been recognized as an essential marker of poor outcomes in older adults with hematological malignancy by clinicians [2]. It is most often defined as an aging-related geriatric syndrome of physiological decline, characterized by significant vulnerability to adverse health outcomes. Frail patients often present an increased age-related impairment in function and physiological capacity, followed by medical complexity and reduced tolerance to medical and surgical interventions. Despite aging, there are other paths that may lead to physical frailty, one of which is chronic disease. Evolving chronic diseases including cancers have been suggested to contribute to the development of physical frailty [5, 6]. The exposures to cancer treatments can also lead to frailty as well [7].

Routine measurement of frailty in hematology practice is feasible, and several measures such as the Geriatric 8 (G8), comprehensive geriatric assessment (CGA) and Clinical Frailty Scale (CFS) are available [8-10]. There is heterogeneity in measuring frailty in hematologic malignancies, with most studies using a Geriatric Assessment (GA) to identify frailty [11]. More recently, studies have reported the predictive value of GA domains in patients with certain types of hematologic malignancies, such as acute myelogenous leukemia (AML) [12], myelodysplastic syndrome (MDS) [12, 13], diffuse large B cell lymphoma (DLBCL) [14, 15], as well as patients who were undergoing hematopoietic cell transplantation (HCT) [16]. However, evidence regarding the association between frailty and clinical outcomes in patients with chronic myeloid leukemia (CML) is limited. In this study, we aimed to evaluate the prevalence, characteristics, and impact of frailty in hospitalized patients with chronic myeloid leukemia (CML) in general and in different age groups, using a nationally representative large cohort of the US.

## Methods

### Data source

This population-based, retrospective study extracted all data from the US Nationwide Inpatient Sample (NIS) database, the largest continuous inpatient care database including about 8 million hospital stays each year [17]. The database is administered by the Healthcare Cost and Utilization Project (HCUP) of the US National Institutes of Health (NIH). Patient data such as patient demographics, procedures, diagnoses, admission and discharge status, duration of hospital stay, and hospital characteristics were obtained. The 2016 HCUP NIS includes all discharge data from 4,573 hospitals. This 2016 NIS sampling frame is comprised of 46 states and the District of Columbia, covering more than 97% of the US population and includes almost 96% of dischargers in the US community hospitals. More details on the design and data framework of the HCUP NIS could be found on: https://health.gov/healthypeople/objectives-and-data/data-sources-and-methods/data-sources/healthcare-cost-and-utilization-project-national-nationwide-inpatient-sample-hcup-nis.

### Ethics statement

All data were obtained from the Online HCUP Central Distributor (https://www.distributor.hcup-us.ahrq.gov/), which administers the database (certificate # HCUP-4T39K81HZ). This study conforms to the NIS data-use agreement with HCUP. Because this study analyzed secondary data from the NIS database, patients and the public were not involved directly. The protocol of this study was submitted and exempted to the Institutional Review Board (IRB) of our Hospital. Due to all data in the NIS database are de-identified, informed consent was also waived.

### Study population

Data of hospitalized patients aged 20 years or older admitted with CML between 2016 and 2018 were identified in the NIS database through the International Classification of Diseases, Tenth Revision, Clinical Modification (ICD-10-CM) codes: C92.10, C92.11, C92.12, C92.20, C92.21, C92.22. Individuals without complete data on main study variables and outcomes were excluded. The cohort was further categorized into patients with or without frailty. The method/criterion for defining frailty is detailed below.

## Study variables

### Study endpoints

Study endpoints were: 1) in-hospital mortality; 2) unfavorable discharge, defined as discharged to a nursing home or long-term facility; 3) prolonged length of stay (LOS) defined as > 75^th^ LOS; and 4) total hospital cost.

### Definition of frailty

To define frailty, we adapted the hospital frailty risk score (HFRS), a previously developed algorithm by Gilbert et al. to identify frailty traits in an electronic database [18]. The HFRS has the advantage of being derived from ICD-10 codes, so it can be used wherever ICD-10 coding systems are in place. This algorithm was validated and increasingly utilized recently in various clinical settings across different countries [19-21]. In the present study, patients who had an HFRS >  = 5 were considered frail, whereas patients with an HRFS < 5 were regarded as non-frail. The codes used to assess HFRS are summarized in Supplementary Table S1.

### Covariates

Data of patients’ demographic characteristics included age, gender, race, household income quartiles, insurance status (primary payer). Household income quartiles were obtained from the NIS, estimated from the household income of residents in the patient's ZIP Code (https://hcup-us.ahrq.gov/db/vars/zipinc_qrtl/nisnote.jsp). Since these estimates are updated annually, the value ranges categories vary by year. The ranges of household income quartiles are summarized in Supplementary Table S4. In addition, individual’s clinical characteristics, including CML status (in remission, not having achieved remission, in relapse), comorbidities, Charlson Comorbidity Index (CCI), and treatments (hematopoietic stem cell transplantation (HSCT) or chemotherapy), were identified using ICD-10 codes. Finally, hospital-related characteristics (bed size, location/teaching status, and hospital region) were also obtained as part of the comprehensive data available for all participants. The codes used to identify the comorbidities, treatments, and CCI are summarized in Supplementary Table S2 and S3.

### Statistical analysis

The NIS database includes a 20% sample of US annual inpatient admissions and as suggested by the guidelines of the database, weighted samples (DISCWT), stratum (NIS_STRATUM), and cluster (HOSPID) were used to derive the national estimates. The SURVEY procedure in SAS performs analysis for sample survey data. Descriptive statistics are presented as number (n) and weighted percentage (%) or mean and standard error (SE). Categorical data was analyzed by PROC SURVEYFREQ statement and continuous data was analyzed by PROC SURVEYREG statement. Logistic regression analyses were performed and determined the associations between study variables and in-hospital mortality, unfavorable discharge, and prolonged LOS. To minimize the differences in baseline characteristics, in the regression analyses, we further excluded 2,057 (15.2%) patients who were in remission and 312 (2.3%) patients in relapse to focus on those not having achieved remission. For the associations between study variables and total hospital cost, natural log-transformed ordinary least squares (OLS) regression were performed to address the potential skewness in distribution of cost. Multivariate regression was adjusted for the significant variables in the univariate regression model. All *p* values are two-sided, and *p* < 0.05 is considered statistically significant. All statistical analyses were performed through SAS software version 9.4 (SAS Institute Inc., Cary, NC, USA). In addition, stratified analyses on the association between frailty and in-hospital mortality by different age groups were also performed.

## Results

During 2016 and 2018, in the NIS database, a total of 13,849 hospitalized CML patients were identified. After exclusion for missing data of sex (*n* = 5), age < 20 (*n* = 176) and no information on study endpoints (*n* = 111), the remaining 13,557 patients were included as the primary cohort. Of them, 49.6% (*n* = 6,719) were frail.

### Baseline characteristics

Baseline characteristics of the study population are summarized in Table 1. Patients’ mean age and HFRS were 65.1 years and 5.6, respectively, and 7,619 (56.2%) were males. As compared with non-frail patients, frail patients were older (69.1 vs. 61.2 years, *p*-value < 0.001), had more females (45.0% vs. 42.6%, *p* = 0.006), had a white race (74.7% vs. 71.2%, *p* < 0.001), with a more significant proportion of insurance covered by medicare/medicaid (80.7% vs. 68.4%, *p* < 0.001), with higher CCI scores (2–3: 32.0% vs. 23.5%; 4 + : 37.2% vs. 16.8%, *p* < 0.001). Significantly higher frequencies of coronary artery disease, congestive heart failure, diabetes, hypertension, cerebrovascular disease, chronic pulmonary disease, drug abuse, severe liver disease, moderate or severe renal disease, and rheumatic disease were observed among frail patients. There were also significant differences in hospital location/teaching status and hospital region between frail and non-frail patients (*p* < 0.001) (Table 1).

**Table 1:** Baseline characteristics of hospitalized CML patients by frailty status

**Study variables**	**Total (*n* = 13,557)**	**Frailty**		***P*-value**
		**Yes (*n* = 6,719)**	**No (*n* = 6,838)**	
HFRS	5.6 ± 0.04	9.0 ± 0.04	2.3 ± 0.02	**< 0.001**
**Demography**				
Age	65.1 ± 0.19	69.1 ± 0.21	61.2 ± 0.24	**< 0.001**
20–39	1235 (9.1)	334 (5.0)	901 (13.2)	**< 0.001**
40–59	3219 (23.7)	1244 (18.5)	1975 (28.9)	
60–79	6277 (46.3)	3313 (49.3)	2964 (43.3)	
80 +	2826 (20.8)	1828 (27.2)	998 (14.6)	
Sex				**0.006**
Male	7619 (56.2)	3694 (55.0)	3925 (57.4)	
Female	5938 (43.8)	3025 (45.0)	2913 (42.6)	
Race				**< 0.001**
White	9567 (72.9)	4853 (74.7)	4714 (71.2)	
Black	1635 (12.5)	776 (11.9)	859 (13.0)	
Hispanic	1123 (8.6)	505 (7.8)	618 (9.3)	
Others	793 (6.0)	366 (5.6)	427 (6.5)	
Missing	439	219	220	
Household income				0.746
Quartile1	3655 (27.4)	1791 (27.0)	1864 (27.8)	
Quartile2	3519 (26.4)	1761 (26.6)	1758 (26.2)	
Quartile3	3303 (24.8)	1658 (25.0)	1645 (24.5)	
Quartile4	2865 (21.5)	1416 (21.4)	1449 (21.6)	
Missing	215	93	122	
Insurance status				**< 0.001**
Medicare/Medicaid	10085 (74.5)	5411 (80.7)	4674 (68.4)	
Private including HMO	2818 (20.8)	1047 (15.6)	1771 (25.9)	
Self-pay/no-charge/other	635 (4.7)	251 (3.7)	384 (5.6)	
Missing	19	10	9	
**Clinical characteristics**				
CML status				0.688
In remission	2057 (15.2)	1008 (15.0)	1049 (15.3)	
Not having achieved remission	11188 (82.5)	5562 (82.8)	5626 (82.3)	
In relapse	312 (2.3)	149 (2.2)	163 (2.4)	
Comorbidities				
Coronary artery disease	3944 (29.1)	2173 (32.3)	1771 (25.9)	**< 0.001**
Congestive heart failure	3862 (28.5)	2332 (34.7)	1530 (22.4)	**< 0.001**
Diabetes	4326 (31.9)	2449 (36.4)	1877 (27.4)	**< 0.001**
Hypertension	4456 (32.9)	1918 (28.5)	2538 (37.1)	**< 0.001**
Cerebrovascular disease	969 (7.1)	732 (10.9)	237 (3.5)	**< 0.001**
Chronic pulmonary disease	3438 (25.4)	1881 (28.0)	1557 (22.8)	**< 0.001**
Obesity	2056 (15.2)	1012 (15.1)	1044 (15.3)	0.744
Drug abuse	1952 (14.4)	816 (12.1)	1136 (16.6)	**< 0.001**
Severe Liver disease	180 (1.3)	114 (1.7)	66 (1.0)	**< 0.001**
Moderate or severe renal disease	4081 (30.1)	2891 (43.0)	1190 (17.4)	**< 0.001**
Rheumatic disease	413 (3.0)	232 (3.5)	181 (2.6)	**0.009**
Long term use of systemic steroid	351 (2.6)	175 (2.6)	176 (2.6)	0.909
CCI				**< 0.001**
0–1	6155 (45.4)	2070 (30.8)	4085 (59.7)	
2–3	3756 (27.7)	2151 (32.0)	1605 (23.5)	
4 +	3646 (26.9)	2498 (37.2)	1148 (16.8)	
Treatment				
Hematopoietic stem cell transplantation	410 (3.0)	190 (2.8)	220 (3.2)	0.188
Chemotherapy	222 (1.6)	46 (0.7)	176 (2.6)	**< 0.001**
**Hospital characteristics**				
Hospital bed size				0.865
Small	2305 (17.0)	1137 (16.9)	1168 (17.1)	
Medium	3678 (27.1)	1837 (27.3)	1841 (26.9)	
Large	7574 (55.9)	3745 (55.7)	3829 (56.0)	
Hospital location/teaching status				**0.001**
Rural	1048 (7.7)	535 (8.0)	513 (7.5)	
Urban nonteaching	2816 (20.8)	1476 (22.0)	1340 (19.6)	
Urban teaching	9693 (71.5)	4708 (70.1)	4985 (72.9)	
Hospital region				**0.002**
Northeast	2638 (19.5)	1241 (18.5)	1397 (20.4)	
Midwest	3144 (23.2)	1639 (24.4)	1505 (22.0)	
South	5399 (39.8)	2644 (39.4)	2755 (40.3)	
West	2376 (17.5)	1195 (17.8)	1181 (17.3)	

Continuous variables are presented as mean ± SE; categorical variables are presented as unweighted counts (weighted percentage)

*HFRS* Hospital Frailty Risk Score, *HMO* health maintenance organization, *CML* chronic myeloid leukemia, *CCI* Charlson Comorbidity Index, *LOS* length of stay. Significant values are shown in bold

### In-hospital outcomes

The mean total hospital cost was 74,773 US dollars. In-hospital mortality, the rate of unfavorable discharge, and prolonged LOS of the study population were 4.4, 18.8, and 29.6%, respectively. In frail patients, greater frequencies of in-hospital death, unfavorable discharge, prolonged LOS, and higher total hospital cost were observed (all *p*-value < 0.001). (Table 2).

**Table 2:** In-hospital outcomes of CML patients by frailty status

**Outcomes**	**Total (*n* = 13,557)**	**Frailty**		***P*-value**
		**Yes (*n* = 6,719)**	**No (*n* = 6,838)**	
In-hospital mortality	603 (4.4)	485 (7.2)	118 (1.7)	**< 0.001**
Unfavorable discharge^a^	2441 (18.8)	1797 (28.8)	644 (9.6)	**< 0.001**
Prolonged LOS^b^	3837 (29.6)	2513 (40.3)	1324 (19.7)	**< 0.001**
Total hospital cost (per USD)	74773.0 ± 1474.6	88448.0 ± 2052.8	61335.0 ± 1555.8	**< 0.001**

Continuous variables are presented as mean ± SE; categorical variables are presented as unweighted counts (weighted percentage)

^a^Patients died in hospital were excluded

^b^Defined as LOS > 75^th^ percentile, i.e., > 7 days

### Associations between in-hospital mortality, unfavorable discharge, prolonged LOS, total hospital cost and frailty

The relationship between frailty and in-hospital mortality, unfavorable discharge, prolonged LOS, and total hospital cost are summarized in Table 3. In multivariate analyses after adjustment, frailty was significantly and independently associated with increased risks for in-hospital mortality (adjusted odds ratio [aOR], 3.81; 95% CI: 2.99–4.86), unfavorable discharge (aOR, 2.90; 95% CI: 2.58–3.27), prolonged LOS (aOR, 2.97; 95% CI: 2.70–3.27), and higher total hospital cost (adjusted beta, 0.33; 95% CI: 0.29–0.37) than non-frailty. (Table 3).

**Table 3:** Associations between frailty and in-hospital mortality, unfavorable discharge, prolonged LOS, and total hospital cost in patients with CML (*n* = 11,188)^c^

Study variable	In-hospital mortality		Unfavorable discharge^a^		Prolonged LOS (> 7 days)^a, b^		Total hospital cost	
	Univariate	Multivariate	Univariate	Multivariate	Univariate	Multivariate	Univariate	Multivariate
	OR (95% CI)	aOR (95% CI)	OR (95% CI)	aOR (95% CI)	OR (95% CI)	aOR (95% CI)	Beta (95% CI)	aBeta (95% CI)
**Frailty (Yes vs no)**	**4.44 (3.56- 5.54)**	**3.81 (2.99- 4.86)**	**3.96 (3.55- 4.41)**	**2.90 (2.58- 3.27)**	**2.74 (2.51- 2.98)**	**2.97 (2.70- 3.27)**	**0.33 (0.29- 0.37)**	**0.36 (0.34- 0.39)**
**Demography**								
Age (vs 20–39)								
40–59	1.37 (0.83- 2.26)	1.36 (0.83- 2.25)	**2.89 (1.91- 4.36)**	**2.26 (1.49- 3.43)**	1.08 (0.91- 1.28)		0.05 (-0.03- 0.14)	**0.05 (0.002- 0.10)**
60–79	**2.55 (1.61- 4.04)**	**2.20 (1.35- 3.58)**	**7.76 (5.23- 11.49)**	**4.35 (2.89- 6.57)**	1.05 (0.90- 1.23)		-0.04 (-0.12- 0.05)	-0.01 (-0.06- 0.05)
80 +	**3.91 (2.45- 6.23)**	**2.65 (1.59- 4.42)**	**19.26 (12.96- 28.62)**	**9.73 (6.39- 14.81)**	1.00 (0.85- 1.19)		**-0.15 (-0.23- -0.06)**	**-0.11 (-0.17- -0.05)**
Sex (Male vs Female)	0.94 (0.79- 1.12)		**1.21 (1.09- 1.33)**	1.06 (0.95- 1.19)	1.00 (0.92- 1.08)		**-0.09 (-0.13- -0.05)**	**-0.07 (-0.09- -0.05)**
Race (vs White)								
Black	0.90 (0.67- 1.19)		**0.81 (0.69- 0.94)**	1.01 (0.84- 1.21)	**1.38 (1.22- 1.57)**	**1.33 (1.16- 1.52)**	**0.07 (0.04- 0.11)**	0.03 (-0.002- 0.07)
Hispanic	0.90 (0.64- 1.26)		**0.48 (0.39- 0.60)**	**0.71 (0.56- 0.90)**	**1.32 (1.14- 1.54)**	**1.35 (1.15- 1.58)**	**0.35 (0.31- 0.39)**	**0.22 (0.18- 0.25)**
Others	0.92 (0.62- 1.37)		**0.55 (0.43- 0.71)**	**0.70 (0.54- 0.91)**	**1.53 (1.26- 1.85)**	**1.59 (1.31- 1.93)**	**0.35 (0.30- 0.41)**	**0.22 (0.17- 0.27)**
Household income (vs Quartile4)								
Quartile1	1.00 (0.77- 1.30)		**0.82 (0.71- 0.95)**		1.00 (0.88- 1.13)		**-0.12 (-0.18- -0.05)**	-0.004 (-0.04- 0.03)
Quartile2	1.11 (0.85- 1.44)		0.92 (0.80- 1.06)		1.09 (0.97- 1.23)		**-0.11 (-0.17- -0.05)**	-0.001 (-0.04- 0.03)
Quartile3	1.09 (0.84- 1.42)		0.93 (0.80- 1.08)		0.97 (0.86- 1.10)		**-0.07 (-0.13- -0.01)**	-0.001 (-0.04- 0.03)
Insurance status (vs Medicare/Medicaid)								
Private including HMO	**0.68 (0.53- 0.87)**	1.11 (0.84- 1.47)	**0.27 (0.23- 0.32)**	**0.54 (0.45- 0.66)**	0.92 (0.82- 1.02)		**0.11 (0.06- 0.17)**	**0.07 (0.04- 0.10)**
Self-pay/no-charge/other	1.18 (0.81- 1.73)	**1.73 (1.16- 2.56)**	**0.27 (0.19- 0.38)**	**0.49 (0.34- 0.70)**	0.88 (0.71- 1.08)		-0.07 (-0.17- 0.03)	**-0.11 (- 0.16- -0.06)**
**Clinical characteristics**								
Comorbidities								
Coronary artery disease	1.00 (0.82- 1.21)		**1.32 (1.19- 1.47)**	**0.77 (0.68- 0.87)**	0.93 (0.85- 1.03)		-0.02 (-0.07- 0.02)	
Congestive heart failure	**1.55 (1.30- 1.86)**	1.10 (0.88- 1.38)	**1.95 (1.76- 2.16)**	1.07 (0.93- 1.23)	**1.43 (1.31- 1.57)**	**1.27 (1.13- 1.42)**	**0.08 (0.03- 0.12)**	**0.09 (0.07- 0.12)**
Diabetes	**0.75 (0.62- 0.91)**	**0.66 (0.52- 0.85)**	**1.39 (1.26- 1.54)**	1.02 (0.90- 1.16)	1.08 (0.99- 1.18)		0.01 (-0.04- 0.05)	
Hypertension	**0.60 (0.49- 0.74)**	**0.58 (0.45- 0.75)**	**0.78 (0.70- 0.87)**	**0.82 (0.71- 0.94)**	**0.74 (0.67- 0.81)**	**0.82 (0.74- 0.92)**	**-0.10 (-0.14- -0.06)**	**-0.05 (-0.07- -0.02)**
Cerebrovascular disease	**2.69 (2.11- 3.42)**	**1.81 (1.37- 2.39)**	**2.61 (2.22- 3.08)**	**1.68 (1.39- 2.04)**	**1.46 (1.25- 1.72)**	1.07 (0.90- 1.28)	**0.26 (0.18- 0.33)**	**0.12 (0.08- 0.17)**
Chronic pulmonary disease	1.17 (0.96- 1.42)		**1.23 (1.10- 1.37)**	0.89 (0.79- 1.02)	1.05 (0.95- 1.16)		-0.02 (-0.06- 0.02)	
Obesity	**0.43 (0.30- 0.62)**	**0.50 (0.35- 0.72)**	1.06 (0.92- 1.21)		**1.15 (1.03- 1.29)**	**1.17 (1.03- 1.33)**	**0.06 (0.01- 0.11)**	**0.07 (0.03- 0.10)**
Drug abuse	**0.60 (0.45- 0.81)**	0.77 (0.56- 1.05)	**0.62 (0.53- 0.73)**	1.06 (0.89- 1.25)	0.96 (0.85- 1.08)		**-0.06 (-0.11- -0.01)**	**-0.05 (-0.08- -0.02)**
Severe Liver disease	**2.42 (1.42- 4.13)**	**2.13 (1.19- 3.82)**	1.21 (0.80- 1.83)		**1.87 (1.31- 2.66)**	**1.67 (1.13- 2.46)**	**0.41 (0.24- 0.58)**	**0.30 (0.20- 0.39)**
Moderate or severe renal disease	**1.39 (1.16- 1.66)**	**0.65 (0.50- 0.85)**	**1.90 (1.71- 2.10)**	**0.65 (0.55- 0.77)**	**1.35 (1.23- 1.48)**	**0.81 (0.70- 0.94)**	**0.04 (0.00- 0.08)**	**-0.11 (-0.15- -0.08)**
Rheumatic disease	0.82 (0.46- 1.43)		**1.31 (1.01- 1.71)**	0.98 (0.73- 1.32)	**1.36 (1.07- 1.72)**	**1.39 (1.07- 1.79)**	0.02 (-0.08- 0.13)	
Long term use of systemic steroid	0.54 (0.26- 1.13)		0.90 (0.66- 1.24)		**0.72 (0.54- 0.96)**	**0.63 (0.46- 0.86)**	-0.09 (-0.20- 0.02)	
CCI (vs 0–1)								
2–3	**1.77 (1.42- 2.19)**	1.22 (0.93- 1.60)	**2.34 (2.07- 2.64)**	**1.48 (1.26- 1.75)**	**1.20 (1.08- 1.33)**	1.00 (0.88- 1.14)	0.03 (-0.02- 0.08)	**0.04 (0.01- 0.08)**
4 +	**1.85 (1.49- 2.29)**	1.30 (0.89- 1.92)	**2.98 (2.64- 3.36)**	**1.94 (1.55- 2.43)**	**1.63 (1.47- 1.81)**	1.18 (0.99- 1.40)	**0.14 (0.09- 0.19)**	**0.12 (0.08- 0.16)**
Treatment								
Hematopoietic stem cell transplantation	1.31 (0.76- 2.26)		**0.35 (0.21- 0.57)**	0.84 (0.50- 1.41)	**2.87 (2.16- 3.82)**	**3.15 (2.29- 4.32)**	**0.88 (0.68- 1.08)**	**0.78 (0.66- 0.89)**
Chemotherapy	0.45 (0.17- 1.22)		**0.09 (0.04- 0.25)**	**0.29 (0.11- 0.77)**	**1.48 (1.07- 2.04)**	**2.03 (1.43- 2.89)**	**0.65 (0.49- 0.80)**	**0.58 (0.49- 0.67)**
**Hospital characteristics**								
Hospital bed size (vs Large)								
Small	**0.76 (0.59- 0.98)**		**1.20 (1.04- 1.37)**	1.03 (0.89- 1.20)	**0.74 (0.66- 0.84)**	**0.72 (0.63- 0.81)**	**-0.36 (-0.42- -0.30)**	**-0.31 (-0.34- -0.27)**
Medium	0.85 (0.69- 1.05)		**1.20 (1.07- 1.35)**	1.11 (0.97- 1.26)	**0.82 (0.74- 0.90)**	**0.77 (0.69- 0.85)**	**-0.14 (-0.19- -0.09)**	**-0.15 (-0.18- -0.11)**
Hospital location/teaching status (vs Urban teaching)								
Rural	1.17 (0.85- 1.62)		1.05 (0.88- 1.24)		**0.50 (0.42- 0.60)**	**0.49 (0.40–0.59)**	**-0.73 (-0.80- -0.67)**	**-0.68 (-0.72- -0.65)**
Urban nonteaching	0.94 (0.75- 1.17)		1.11 (0.98- 1.25)		**0.80 (0.72- 0.89)**	**0.79 (0.70- 0.88)**	**-0.17 (-0.22- -0.11)**	**-0.15 (-0.18- -0.12)**
Hospital region (vs Northeast)								
Midwest	**0.76 (0.58- 0.99)**		0.96 (0.83- 1.13)	0.93 (0.78- 1.10)	**0.81 (0.71- 0.93)**	**0.78 (0.68- 0.91)**	**-0.24 (-0.32- -0.17)**	**-0.20 (-0.27- -0.14)**
South	**0.75 (0.59- 0.96)**		**0.86 (0.75- 0.99)**	0.88 (0.76- 1.03)	1.08 (0.96- 1.22)	1.09 (0.96- 1.24)	-0.05 (-0.12- 0.02)	-0.03 (-0.09- 0.03)
West	0.84 (0.63- 1.11)		**0.68 (0.57- 0.80)**	**0.67 (0.56- 0.80)**	0.90 (0.78- 1.04)	**0.82 (0.70- 0.96)**	**0.28 (0.20- 0.36)**	**0.23 (0.18- 0.29)**

Significant values were shown in bold

*HMO* health maintenance organization, *CML* chronic myeloid leukemia, *CCI* Charlson Comorbidity Index, *LOS* length of stay, *OLS* ordinary least squares

^a^Patients died in hospital were excluded

^b^LOS > 75th percentile

^b^Utilizing natural log-transformed OLS regression

^c^Excluding 2,057 (15.2%) patients in remission and 312 (2.3%) patients in relapse

### Association between frailty and in-hospital mortality stratified by age

Table 4 shows the relationship between frailty and in-hospital mortality stratified by age. After adjusting by insurance status, CML status, congestive heart failure, diabetes, hypertension, cerebrovascular disease, obesity, drug abuse, severe liver disease, moderate or severe renal disease, CCI, hospital bed size, and hospital region, frailty remained significantly associated with more significant risks for in-hospital mortality in all age -groups, while the most significant risk was observed among patients aged 40–59 years old (aOR, 5.90; 95% CI: 3.08–11.31) (Table 4).

**Table 4:** Association between frailty and in-hospital mortality in patients with CML stratified by age (*n* = 11,188)^b^

Age, years	In-hospital mortality			
	Univariate		Multivariate	
	OR (95% CI)	*p*-value	aOR^a^ (95% CI)	*p*-value
20–39	**5.47 (2.25- 13.27)**	**< 0.001**	**3.71 (1.10- 12.55)**	**0.035**
40–59	**7.21 (4.08- 12.76)**	**< 0.001**	**5.90 (3.08- 11.31)**	**< 0.001**
60–79	**3.02 (2.27- 4.01)**	**< 0.001**	**2.95 (2.16- 4.03)**	**< 0.001**
80 +	**4.25 (2.72- 6.64)**	**< 0.001**	**4.16 (2.63- 6.58)**	**< 0.001**

Significant values are shown in bold

^a^Adjusted for insurance status, congestive heart failure, diabetes, hypertension, cerebrovascular disease, obesity, drug abuse, severe liver disease, moderate or severe renal disease, and CCI

^b^Excluding 2,057 (15.2%) patients in remission and 312 (2.3%) patients in relapse

## Discussion

Half of hospitalized CML patients in the US were frail defined by HFRS. Frailty is significantly and independently associated with nearly 4-time risk of in-hospital mortality, 3-time of unfavorable discharge, 3-time of prolonged LOS, and significantly higher cost during admission. Furthermore, the impact of frailty on in-hospital death is seen in both elderly and non-elderly patients. These findings indicate that frailty is a strong independent predictor for adverse in-patient outcomes in hospitalized CML patients.

A review study by Handforth et al. reported 42 and 43% prevalence of frailty and pre-frailty in older adults with solid tumor and hematological malignancy, respectively [22]. Atakul et al. reported a frailty prevalence of 42.2% in older patients undergoing treatment for hematological malignancies [23]. Patel et al. reported a 41.3 and 29.9% prevalence of prefrailty and frailty [24]. The present study found a higher prevalence of frailty (52.8%) in CML patients aged over 60 than in the previous studies. It may be explained by this study utilized an acute care cohort, thereby the prevalence of frailty is likely to be higher.

Studies showed frailty can provide a better measure of vulnerability of worse outcomes than age in hematologic malignancies. Facon et al. investigated the outcomes of patients from the large, phase 3 FIRST trial in newly diagnosed multiple myeloma (NDMM) based on frailty using scores for age, Charlson Comorbidity Index (CCI), and Eastern Cooperative Oncology Group performance status (ECOG PS) [25]. The authors of that study concluded simplified frailty scale predicts worse progression-free and overall survival among transplant-ineligible patients with newly diagnosed multiple myeloma [25]. Abel et al. reviewed the outcomes of different blood cancers in patients by the methods of Vulnerable Elders Survey (VES-13), G8, Geriatric Assessment in Hematology (GAH), CFS, Timed Up and Go (TUG), and International Myeloma Working Group (IMWG) Frailty Score, indicating that frailty assessment rather than age could help hematologist in practice [10]. Scheepers et al. suggested older patients with hematologic malignancy with geriatric impairments had a higher risk of treatment-related toxicity, treatment non-completion, and healthcare services utility, indicating that frailty assessments should be considered before starting treatment in older patients with hematologic malignancies.

Individuals with a cancer are dealing with the interacting effects of the biologic and physiologic changes of aging, multimorbidity, effects of the cancer per se, and the effects of the cancer treatments, among which chemotherapy has the most pervasive effect [26]. It was also documented that frailty in blood cancers may originate from various conditions including cancer issues (e.g., weight loss, cachexia), comorbidities, immunosuppression, or treatment-related toxicity [27]. In particular, high-intensity therapeutic exposures, chronic graft-versus-host disease (GvHD), and chronic health conditions after hematopoietic stem cell transplantation (HSCT) serve as substantial stressors, increasing the risk of frailty even among nonelderly [28]. Of note, the proportion of patients who received chemotherapy or HSCT in this study cohort was very low, probably due to not properly coded in the database.

Many molecular targeting drugs have been used in the clinic and might have the potential to replace conventional chemotherapy and HSCT [29]. For example, tyrosine kinase inhibitors (TKIs), such as imatinib or dasatinib, are thought to be the mainstay of CML treatment and drastically improved outcomes of CML [29–31]. Treatment of CML with TKIs results in near-normal life expectancy. However, studies have reported that TKI may lead to skeletal muscle loss in cancer treatment, affecting patients' health with more extended treatment [30–32]. The NIS database does not provide information on medication used, which hindered further evaluation on the potential causal influence between TKIs and frailty. Since there is currently no data regarding whether and how TKIs interact with frailty in patients with hematologic cancers, it is important to conduct such investigations in the future.

The present study included non-elderly CML patients. It is demonstrated that the prevalence of frailty is 27 and 38.6% in patients aged 20–39 years and 40–59 years, respectively. Importantly, we found that frailty is strongly associated with increased risk for in-hospital mortality not only in elderly patients but also in the non-elderly. In young CML patients, it is postulated that frailty is more likely related to CML treatments. Future studies focused on the origin and impact of frailty in non-elderly CML patients is warranted.

### Strengths and limitations

The strength of the present study is the use of a very large sample that represents a nationwide population. One of the major limitations was that we were not able to distinguish disease-related frailty, which may be a consequence of CML and its treatment, from age-related frailty. HFRS has yet to be formally validated in population under 75 years old as well as in cancer patients. Nevertheless, a list of recent studies did have assessed the prognostic role of HFRS and expanded its coverage to younger population aged 20–75 years [33, 34]. Other limitations include the possibility of coding errors during use of the ICD-10 coding systems for defining CML, comorbidities and complications. Possible confounding variables not collected by the NIS could not be included in the analyses. Targeted therapies are crucial for the management of CML. However, they could not be captured through the coding system thus could not be analyzed. The study also lacks follow-up data after discharge, precluding the evaluation of late morbidity and mortality.

## Conclusions

Frailty is a strong predictor for increased in-hospital mortality, unfavorable discharge, prolonged LOS, and more hospital cost in CML patients in the US. Frailty not only poses greater risk for in-hospital death in older patients but also in non-elderly. Future studies that include data of targeted therapies are warranted.

## Supplementary Information

**Additional file 1:** **Supplementary Table S1.** ICD-10 codes used to assess HFRS. **Supplementary Table S2.** ICD-10 codes used to identify CML, comorbidities and treatments. **Supplementary Table S3.** ICD-10 codes used to define CCI. **Supplementary Table S4.** Quartile ranges of household income (USD).

## Data Availability

All data analysed during this study are included in this published article.

## Abbreviations

CML: Chronic myeloid leukemia
NIS: Nationwide Inpatient Sample
ICD: ICD-10International Classification of Diseases
CGA: Comprehensive geriatric assessment
CFS: Clinical Frailty Scale
AML: Acute myelogenous leukemia
MDS: Myelodysplastic syndrome
DLBCL: Diffuse large B cell lymphoma
HCT: Hematopoietic cell transplantation
HCUP: Healthcare Cost and Utilization Project
NIH: National Institutes of Health
