# Supplementary material for: Frailty in chronic myeloid leukemia: evidence from 2016–2018 Nationwide Inpatient Sample of the US
Source: BMC Geriatr. 2023 May 30;23:334. doi: 10.1186/s12877-023-03962-7 (PMC10228104; doi:10.1186/s12877-023-03962-7)
Supplement: Supplementary file 1 — Additional file 1: Supplementary Table S1. ICD-10 codes used to assess HFRS. Supplementary Table S2. ICD-10 codes used to identify CML, comorbidities and treatments. Supplementary Table S3. ICD-10 codes used to define CCI. Supplementary Table S4. Quartile ranges of household income (USD). [file 12877_2023_3962_MOESM1_ESM.docx]

**Supplementary Table S1. ICD-10 codes used to assess HFRS.**

| Condition | ICD-10-CM | Points |
| --- | --- | --- |
| Dementia in Alzheimer's disease | F00 | 7.1 |
| Hemiplegia | G81 | 4.4 |
| Alzheimer's disease | G30 | 4.0 |
| Sequelae of cerebrovascular disease (secondary codes) | I69 | 3.7 |
| Other symptoms and signs involving the nervous and musculoskeletal systems (R29·6 Tendency to fall) | R29 | 3.6 |
| Other disorders of urinary system (includes urinary tract infection and urinary incontinence) | N39 | 3.2 |
| Delirium, not induced by alcohol and other psychoactive substances | F05 | 3.2 |
| Unspecified fall | W19 | 3.2 |
| Superficial injury of head | S00 | 3.2 |
| Unspecified haematuria | R31 | 3.0 |
| Other bacterial agents as the cause of diseases classified to other chapters (secondary code) | B96 | 2.9 |
| Other symptoms and signs involving cognitive functions and awareness | R41 | 2.7 |
| Abnormalities of gait and mobility | R26 | 2.6 |
| Other cerebrovascular diseases | I67 | 2.6 |
| Convulsions, not elsewhere classified | R56 | 2.6 |
| Somnolence, stupor and coma | R40 | 2.5 |
| Complications of genitourinary prosthetic devices, implants and grafts | T83 | 2.4 |
| Intracranial injury | S06 | 2.4 |
| Fracture of shoulder and upper arm | S42 | 2.3 |
| Other disorders of fluid, electrolyte and acid- base balance | E87 | 2.3 |
| Other joint disorders, not elsewhere classified | M25 | 2.3 |
| Volume depletion | E86 | 2.3 |
| Senility | R54 | 2.2 |
| Care involving use of rehabilitation procedures | Z50 | 2.1 |
| Unspecified dementia | F03 | 2.1 |
| Other fall on same level | W18 | 2.1 |
| Problems related to medical facilities and other health care | Z75 | 2.0 |
| Vascular dementia | F01 | 2.0 |
| Superficial injury of lower leg | S80 | 2.0 |
| Cellulitis | L03 | 2.0 |
| Blindness and low vision | H54 | 1.9 |
| Deficiency of other B group vitamins | E53 | 1.9 |
| Problems related to social environment | Z60 | 1.8 |
| Parkinson's disease | G20 | 1.8 |
| Syncope and collapse | R55 | 1.8 |
| Fracture of rib(s), sternum and thoracic spine | S22 | 1.8 |
| Other functional intestinal disorders | K59 | 1.8 |
| Acute renal failure | N17 | 1.8 |
| Decubitus ulcer | L89 | 1.7 |
| Carrier of infectious disease | Z22 | 1.7 |
| Streptococcus and staphylococcus as the cause of diseases classified to other chapters | B95 | 1.7 |
| Ulcer of lower limb, not elsewhere classified | L97 | 1.6 |
| Other symptoms and signs involving general sensations and perceptions | R44 | 1.6 |
| Duodenal ulcer | K26 | 1.6 |
| Hypotension | I95 | 1.6 |
| Unspecified renal failure | N19 | 1.6 |
| Other septicaemia | A41 | 1.6 |
| Personal history of other diseases and conditions | Z87 | 1.5 |
| Respiratory failure, not elsewhere classified | J96 | 1.5 |
| Exposure to unspecified factor | X59 | 1.5 |
| Other arthrosis | M19 | 1.5 |
| Epilepsy | G40 | 1.5 |
| Osteoporosis without pathological fracture | M81 | 1.4 |
| Fracture of femur | S72 | 1.4 |
| Fracture of lumbar spine and pelvis | S32 | 1.4 |
| Other disorders of pancreatic internal secretion | E16 | 1.4 |
| Abnormal results of function studies | R94 | 1.4 |
| Chronic renal failure | N18 | 1.4 |
| Retention of urine | R33 | 1.3 |
| Unknown and unspecified causes of morbidity | R69 | 1.3 |
| Other disorders of kidney and ureter, not elsewhere classified | N28 | 1.3 |
| Unspecified urinary incontinence | R32 | 1.2 |
| Other degenerative diseases of nervous system, not elsewhere classified | G31 | 1.2 |
| Nosocomial condition | Y95 | 1.2 |
| Other and unspecified injuries of head | S09 | 1.2 |
| Symptoms and signs involving emotional state | R45 | 1.2 |
| Transient cerebral ischaemic attacks and related syndromes | G45 | 1.2 |
| Problems related to care-provider dependency | Z74 | 1.1 |
| Other soft tissue disorders, not elsewhere classified | M79 | 1.1 |
| Fall involving bed | W06 | 1.1 |
| Open wound of head | S01 | 1.1 |
| Other bacterial intestinal infections | A04 | 1.1 |
| Diarrhoea and gastroenteritis of presumed infectious origin | A09 | 1.1 |
| Pneumonia, organism unspecified | J18 | 1.1 |
| Pneumonitis due to solids and liquids | J69 | 1.0 |
| Speech disturbances, not elsewhere classified | R47 | 1.0 |
| Vitamin D deficiency | E55 | 1.0 |
| Artificial opening status | Z93 | 1.0 |
| Gangrene, not elsewhere classified | R02 | 1.0 |
| Symptoms and signs concerning food and fluid intake | R63 | 0.9 |
| Other hearing loss | H91 | 0.9 |
| Fall on and from stairs and steps | W10 | 0.9 |
| Fall on same level from slipping, tripping and stumbling | W01 | 0.9 |
| Thyrotoxicosis [hyperthyroidism] | E05 | 0.9 |
| Scoliosis | M41 | 0.9 |
| Dysphagia | R13 | 0.8 |
| Dependence on enabling machines and devices | Z99 | 0.8 |
| Agent resistant to penicillin and related antibiotics | U80 | 0.8 |
| Osteoporosis with pathological fracture | M80 | 0.8 |
| Other diseases of digestive system | K92 | 0.8 |
| Cerebral Infarction | I63 | 0.8 |
| Calculus of kidney and ureter | N20 | 0.7 |
| Mental and behavioural disorders due to use of alcohol | F10 | 0.7 |
| Other medical procedures as the cause of abnormal reaction of the patient | Y84 | 0.7 |
| Abnormalities of heart beat | R00 | 0.7 |
| Unspecified acute lower respiratory infection | J22 | 0.7 |
| Problems related to life-management difficulty | Z73 | 0.6 |
| Other abnormal findings of blood chemistry | R79 | 0.6 |
| Personal history of risk-factors, not elsewhere classified | Z91 | 0.5 |
| Open wound of forearm | S51 | 0.5 |
| Depressive episode | F32 | 0.5 |
| Spinal stenosis (secondary code only) | M48 | 0.5 |
| Disorders of mineral metabolism | E83 | 0.4 |
| Polyarthrosis | M15 | 0.4 |
| Other anaemias | D64 | 0.4 |
| Other local infections of skin and subcutaneous tissue | L08 | 0.4 |
| Nausea and vomiting | R11 | 0.3 |
| Other noninfective gastroenteritis and colitis | K52 | 0.3 |
| Fever of unknown origin | R50 | 0.1 |

ICD-10, International Classification of Disease 10th Revision; HFRS, hospital frailty risk score.

**Supplementary Table S2. ICD-10 codes used to identify CML, comorbidities and treatments.**

|  | ICD-10-CM / ICD-10-PCS |
| --- | --- |
| **CML** |  |
| In remission | C92.11, C92.21 |
| Not having achieved remission | C92.10, C92.20 |
| In relapse | C92.12, C92.22 |
| **Comorbidities** |  |
| Coronary artery disease | I25 |
| Congestive heart failure | I09.9, I11.0, I13.0, I13.2, I25.5, I42.0, I42.5-I42.9, I43.x, I50.x, P29.0 |
| Diabetes | E10-E14 |
| Hypertension | I10 |
| Cerebrovascular disease | G45.x, G46.x, H34.0, I60.x-I69.x |
| Chronic pulmonary disease | I27.8, I27.9, J40.x-J47.x, J60.x-J67.x, J68.4, J70.1, J70.3 |
| Obesity | E66.0-E66.2, E66.8, E66.9, Z68.3-Z68.4 |
| Drug abuse | Z71.5, F10-F19 |
| Severe Liver disease | I85.0, I85.9, I86.4, I98.2, K70.4, K71.1, K72.1, K72.9, K76.5, K76.6, K76.7 |
| Moderate or severe renal disease | I12.0, I13.1, N03.2-N03.7, N05.2-N05.7, N18.x, N19.x, N25.0, Z49.0-Z49.2, Z94.0, Z99.2 |
| Rheumatic disease | M05.x, M06.x, M31.5, M32.x-M34.x, M35.1, M35.3, M36.0 |
| Long term use of systemic steroid | Z79.52 |
| **Treatments** |  |
| HSCT | Z94.81, T86.0; ICD10-PCS:30230G2, 30230G3, 30230Y2, 30230Y3, 30233G2, 30233G3, 30233Y2, 30233Y3, 30240G2, 30240G3, 30240Y2, 30240Y3, 30243G2, 30243G3, 30243Y2, 30243Y3; 30230C0, 30230G0, 30233C0, 30233G0, 30240C0, 30240G0, 30243C0, 30243G0, 30230Y0, 30233Y0, 30240Y0, 30243Y0 |
| Chemotherapy | Z51.11 |

ICD-10, International Classification of Disease 10th Revision; HCST, hematopoietic stem cell transplantation

**Supplementary Table S3. ICD-10 codes used to define CCI.**

|  | ICD-10-CM | Score |
| --- | --- | --- |
| Myocardial infarction | I21.x, I22.x, I25.2 | 1 |
| Congestive heart failure | I09.9, I11.0, I13.0, I13.2, I25.5, I42.0, I42.5-I42.9, I43.x, I50.x, P29.0 | 1 |
| Peripheral vascular disease | I70.x, I71.x, I73.1, I73.8, I73.9, I77.1, I79.0, I79.2, K55.1, K55.8, K55.9, Z95.8, Z95.9 | 1 |
| Cerebrovascular disease | G45.x, G46.x, H34.0, I60.x-I69.x | 1 |
| Dementia | F00.x-F03.x, F05.1, G30.x, G31.1 | 1 |
| Chronic pulmonary disease | I27.8, I27.9, J40.x-J47.x, J60.x-J67.x, J68.4, J70.1, J70.3 | 1 |
| Rheumatic disease | M05.x, M06.x, M31.5, M32.x-M34.x, M35.1, M35.3, M36.0 | 1 |
| Peptic ulcer disease | K25.x-K28.x | 1 |
| Mild liver disease (without portal hypertension, includes chronic hepatitis) | B18.x, K70.0-K70.3, K70.9, K71.3-K71.5, K71.7, K73.x, K74.x, K76.0, K76.2-K76.4, K76.8, K76.9, Z94.4 | 1 |
| Diabetes without chronic complication | E10.0, E10.l, E10.6, E10.8, E10.9, E11.0, E11.1, E11.6, E11.8, E11.9, E12.0, E12.1, E12.6, E12.8, E12.9, E13.0, E13.1, E13.6, E13.8, E13.9, E14.0, E14.1, E14.6, E14.8, E14.9 | 1 |
| Diabetes with chronic complication | E10.2-E10.5, E10.7, E11.2-E11.5, E11.7, E12.2-E12.5, E12.7, E13.2-E13.5, E13.7, E14.2-E14.5, E14.7 | 2 |
| Hemiplegia or paraplegia | G04.1, G11.4, G80.1, G80.2, G81.x, G82.x, G83.0-G83.4, G83.9 | 2 |
| Moderate or severe renal disease | I12.0, I13.1, N03.2-N03.7, N05.2-N05.7, N18.x, N19.x, N25.0, Z49.0-Z49.2, Z94.0, Z99.2 | 2 |
| Moderate or severe liver disease | I85.0, I85.9, I86.4, I98.2, K70.4, K71.1, K72.1, K72.9, K76.5, K76.6, K76.7 | 3 |
| Metastatic solid tumor | C77.x-C80.x | 6 |
| AIDS | B20.x-B22.x, B24.x | 6 |

CML, chronic myeloid leukemia; ICD-10, International Classification of Disease 10th Revision; CCI, Charlson Comorbidity Index.

**Supplementary Table S4. Quartile ranges of household income (USD).**

| Year | Quartile 1 | Quartile 2 | Quartile 3 | Quartile 4 |
| --- | --- | --- | --- | --- |
| 2016 | 1 - 42,999 | 43,000 - 53,999 | 54,000 - 70,999 | 71,000+ |
| 2017 | 1 - 43,999 | 44,000 - 55,999 | 56,000 - 73,999 | 74,000+ |
| 2018 | 1 - 45,999 | 46,000 - 58,999 | 59,000 - 78,999 | 79,000+ |
